# *Paecilomyces formosus* MD12, a Biocontrol Agent to Treat *Meloidogyne incognita* on Brinjal in Green House

**DOI:** 10.3390/jof7080632

**Published:** 2021-08-03

**Authors:** Alaa Baazeem, Mohammed Alorabi, Palanisamy Manikandan, Saqer S. Alotaibi, Abdulaziz Almanea, Ahmed Abdel-Hadi, Ponnuswamy Vijayaraghavan, Subhanandharaj Russalamma Flanet Raj, Young Ock Kim, Hak-Jae Kim

**Affiliations:** 1 Department of Biology, College of Science, Taif University, P.O. Box 11099, Taif 21944, Saudi Arabia; aabaazeem@tu.edu.sa; 2 Department of Biotechnology, College of Science, Taif University, P.O. Box 11099, Taif 21944, Saudi Arabia; morabi@tu.edu.sa (M.A.); saqer@tu.edu.sa (S.S.A.); 3 Department of Medical Laboratory Sciences, College of Applied Medical Sciences, Majmmah University, Majmaah 11952, Saudi Arabia; m.palanisamy@mu.edu.sa; 4 Greenlink Analytical and Research Laboratory (India) Private Limited, Coimbatore 641014, India; 5 Section of Microbiology, Department of Laboratory, King Saud Hospital, P.O. Box 7, Unaizah 51911, Saudi Arabia; aalmanea@moh.gov.sa; 6 Botany and Microbiology Department, Faculty of Science, Al-Azhar University, Assiut Branch, Assiut 71524, Egypt; 7 Bioprocess Engineering Division, Smykon Biotech Private Limited, Nagercoil, Kanyakumari 629201, India; venzymes@gmail.com; 8 Department of PG Zoology, Nesamony Memorial Christian College, Kanyakumari 629165, India; flanetrajsr@gmail.com; 9 Department of Clinical Pharmacology, College of Medicine, Soonchunhyang University, Cheonan 31151, Korea; kyo9128abcd@gmail.com (Y.O.K.); hak3962@sch.ac.kr (H.-J.K.)

**Keywords:** fungal toxins, biocontrol, antagonistic, nematode, root-knot, growth promoters

## Abstract

The present study was carried out to analyze the potential of fungi isolated from the rhizosphere of soybean, brinjal, tomato, and potato plants. The density of fungi varied in the pot soil and rhizosphere after *Paecilomyces formosus* MD12 treatment. The *P. formosus* MD12 population was 6.3 ± 0.13 × 10^4^ CFU g^−1^ in the pot planted with brinjal, and the population increased in the rhizosphere (6.72 ± 0.41 × 10^4^ CFU g^−1^). *P. formosus* MD12 was cultured in the production medium, and the supernatant was used for egg inhibition studies on a root-knot nematode parasite, *Meloidogyne incognita*. It was revealed that maximum egg inhibition (94.7 ± 6.2%) was obtained at 100% concentration of extract. The culture supernatant from *P. formosus* MD12 affected the development of *M. incognita* juvenile, and the mortality rate was maximum after 96 h (95 ± 6%). Mortality was reduced when treated with 25%, 50%, and 75% supernatant. At 1 × 10^7^ mL^−1^ of spore suspension, we found reductions of 71.6 ± 3.3% nematode populations in the soil, 60.7 ± 2.2% from the root, and 63.6 ± 2.4% egg mass compared with the control in the pot experiment. The culture supernatant applied at the 10% level showed a maximum mean reduction of the nematode population in roots (72.4 ± 2.2%), soil (77.9 ± 2.5%), and egg masses (73.2 ± 1.5%), respectively. The presence of *P. formosus* MD12 in a soil environment could antagonize nematode parasites and improve soil amendment. The *P. formosus* MD12 strain showed good biocontrol ability against the root-knot nematode, *M. incognita*, under in vitro and green house experimental condition.

## 1. Introduction

In plants, *Paecilomyces* plays a potent role as an endophytic fungus by providing various advantages for the development of plants. It can be indirectly or directly used as an effective biostimulant. The metabolites of *Paecilomyces* or the organism increases the crop yield and enhances the morphological parameters. Fungus from the genus *Paecilomyces* has irregularly branched conidiophores, hyaline to yellowish septate hyphae, and phialides with an elongated neck and wide base. The youngest conidium occupies the basal end, while conidia are in chains, hyaline and unicellular. The thermo-tolerance nature of conidia was associated with shape and size. Thus, the spherical asexual or smaller conidia are highly vulnerable to high temperature [1,2,3]. *Paecilomyces* grows over various substrates and at various temperature ranges and shows increased sporulation. As a result, its affordable growth on various substrates and rapid multiplication ensures commercial applications in agriculture [4]. *Paecilomyces* has various species; both saprophytic and pathogenic can be occupied in different habitats, including food, decomposing plant material, soil, nematodes, insects, compost, marine sediments, pasteurized foods and products, nematodes, and the rhizosphere of various plants [5,6,7,8]. 

The association of *Paecilomyces* and plants effectively provides protection from phytopathogens and improves plant health by various molecular mechanisms. This symbiotic interaction revealed the production of various phytohormones, including indoleacetic acid and gibberellins that mitigated the influences of abiotic stress such as salinity and promoted plant growth [9,10]. When the fungus, *Paecilomyces*, is administered with pathogenic nematodes, *Paecilomyces* shows toxic effect to the nematodes and improved crop growth and acts as a biological control agent [11,12]. Many species of *Paecilomyces* produce various secondary metabolites with novel biological activities such as cytotoxic, nematicidal, fungicidal, bactericidal, insecticidal, and herbicidal [13]. The secondary metabolites produced by *Paecilomyces* sp. showed enzyme inhibitory and antitumor activity [14].

*Paecilomyces* species are found in alkaline and acidic environments and tolerate various environmental conditions. They severely cause the deterioration of paper, food, and grain and produce mycotoxins. This organism contaminated ground red pepper, herbs, and spices, producing mycotoxins highly harmful to animals [15]. Recent studies have revealed evidence of the serious risk of long-term storage of seeds affected with various *Paecilomyces* sp. *Paecilomyces variotii* is one of the important species involved in contamination or food spoilage. It has been reported from the pasteurized beverages and causes severe economic loss. This organism can also be reported in seed, oils, nuts, meat products, fruits, grain/cereals, dried fruits, and processed cheeses [16]. This fungal strain has the ability to degrade the food preservatives such as propionic acid, benzoic acid, and sorbic acid, and it also induced odor in food products [17].

Pathogenic fungi have various properties that make them highly useful as biological control agents, as they can usually penetrate through the cuticles of insects and increase the epizootics and enhance mortality rates in the pests. These fungi follow an asymptomatic mode of life and can colonize plant tissues as endophytes. Endophytic fungi of the genera *Isaria, Verticillium, Lecanicillium, Paecilomyces, Clonostachys, Cladosporium, Beauveria,* and Acremonium have been isolated from various economic important crop species such asbean (*Phaseolus vulgaris* L.), lettuce (*Lactuca sativa* L.), cocoa (*Theobroma cacao* L.), rice (*Oryza sativa* L.), date palm (*Phoenix dactylifera* L.), banana (*Musa paradisiaca* L.), tomato (*Lycopersiconesculentum* Mill.), cotton (*Gossypium hirsutum* L.), corn (*Zea mays* L.), potato (*Solanum tuberosum* L.), and coffee (*Coffeaarabica* L.) [18,19,20]. In the case of microorganisms, the composition of culture medium critically affects the yield and virulence of biocontrol substances against phyto pathogens [21]. The optimum composition of the nutrient medium increased sporulation and enhanced the growth of fungal mycelia [22]. 

Fungi use various substrates as nitrogen and carbon sources [23]. Moreover, simple and low-cost culture media will be a good choice for the production of microbial mass and secondary metabolites. For the successful application of natural pesticides from the fungus for the application of biocontrol, it is very important to produce better yields of propagules, which are highly pathogenic toward target bacterial and fungal pathogens. The quantity as well as quality of metabolites is significantly affected by culture condition and medium compositions [24]. The ratio of nitrogen–carbon as well as sugar in culture media plays an effective role in determining the pathogenicity of the organism [21]. Hence, carbon and nitrogen sources are frequently screened for the production of secondary metabolites because they may stimulate the production of biocontrol agents. Screening of fungal bioagents for analyzing their ability to control nematode growth in vitro is considered as the suitable method for the analysis of nematocidal properties. The scope of this study was to analyze the ability of indigenous pathogenic fungus and study the inhibitory property on a root-knot nematode parasite, *Meloidogyne incognita*.

## 2. Materials and Methods

### 2.1. Isolation and Identification of Paecilomyces from the Rhizosphere 

Roots of soybean, tomato, cucumber, corn, brinjal, mustard, cow pea, and onion plants were collected and the surface was thoroughly cleaned by running tap water. After the complete removal of soil and debris from the root, it was air dried and used for the isolation of various endophytic fungi. The root samples were placed in a 5% sodium hypochlorite solution (10 mL) for 10 min in a shaker at 75 rpm/min. Then, the surface of the root was washed three times with sterile water to remove sodium hypochlorite solution. The root was further cut into 1–2 cm long pieces, and the roots were macerated with sterile sodium chloride solution (0.9%, *w*/*v*). The homogenized root sample was placed onto potato dextrose agar medium (PDA) incorporated with chloramphenicol (50 µg mL^−1^) to inhibit the growth of bacteria. It was incubated for 7 days at 25 ± 1 °C. The isolated hyphal tip of the developing young colonies was placed on a PDA culture medium and incubated at 25 ± 1 °C for 7 days. The colony morphology of the isolated strain was observed on PDA medium, and the morphology of the conidiophore was determined using light microscopy. The isolated strains were maintained on PDA slants, and subculture was made periodically. Based on microscopic identification, a fungus from the genus *Paecilomyces* was used for this study. The selected strain was identified by molecular methods as suggested by Al-Dhabi et al. [25]. The genomic DNA of MD12 was isolated using theInsta GeneTM Matrix Genomic DNA isolation kit. The ITS region was amplified using ITS1 (TCCGTAGGTGAACCTGCGG) and ITS4 (TCCTCCGCTTATTGATATGC) primers and amplified using MJ Research PTC-225 Peltier Thermal Cycler. The amplified gene product was sequenced using ABI 3730xl sequencer (Applied Biosystems, Waltham, MA, USA). The 18S rDNA was subjected to similarity search using the BLAST search tool and identified as *Paecilomyces formosus* MD12. The phylogenetic relationship between the MD12 sequence and closely related sequence was analyzed by Neighbor-Joining method. The 18S rDNA gene sequence was submitted, and a GenBank accession number was assigned (MZ497411).

### 2.2. Spore Suspension Preparation

The selected fungal strain *P. formosus* MD12 was grown on PDA medium and was incubated for two weeks at 25 ± 2 °C under dark. The culture was scraped on the surface of the agar after 14 days, and conidial medium suspension was prepared. It was transferred into sterile double-distilled water containing Tween 80 (0.05%). The mixture of hyphae and spore was stirred for 15 min, and the hyphae were removed after filtration. The concentration of conidia was analyzed using a hemocytometer and maintained at 1 × 10^6^ mL^−1^. The conidia were diluted appropriately with double-distilled water containing Tween 80 (0.1%, *v*/*v*) and stored at 4 °C until required. Analysis of an in vitro nematode test was performed to find the efficacy of selected fungal spores against nematode parasite. The conidia suspension was diluted at various concentrations (1 × 10^4^–1 × 10^7^ CFU mL^−1^), and the conidia were applied directly to the nematode eggs [3].

### 2.3. Cell Free Supernatant and Nematode Bioassay

The cell-free extract of *P. formosus* MD12 was prepared using Sabouraud dextrose yeast medium. The medium pH was adjusted to 5.5 ± 0.5 and sterilized. After complete sterilization, the Erlenmeyer flask was inoculated with fungal spores and incubated for 14 days at 25 °C. After 2 weeks, the supernatant was filtered using Whatman filter paper No.1, and the mycelial mat was removed. Then, the filtered sample was diluted appropriately and used for bioassay against juvenile nematode parasite [11].

### 2.4. Analysis of Fungal Densities in Rhizosphere and Soil

Fungi from the rhizosphere of selected plants were isolated as described in Section 2.1. To determine the fungal densities in the soil sample, sampling was performed using a cork borer a 3 cm distance from the plant. About 2 g of soil sample was transferred to an Erlenmeyer flask containing double-distilled water (50 mL). It was shaken with an orbital shaker at 150 rpm. Then, it was diluted appropriately and plated on PDA plates. After 5 days incubation at 25 ± 2 °C, the developed fungal colonies were numbered, and CFUg^−1^ dry soil was calculated. The plant was uprooted, and the associated soil was collected after the gentle removal of loosely adhered soil from the root [12].

### 2.5. Analysis of Endophytic Colonization of Fungi

To determine endophytic colonization study, seeds (tomato, cucumber, soybean, corn, brinjal, mustard, cow pea, and onion) were soaked in a conidial suspension (1 × 10^4^ conidia/mL) for 4 h. Sterile double-distilled water was used to soak the brinjal seed and considered as the control. Seeds were air dried for 10 min and carefully transferred in a pot containing plating material. The substrate (red soil and earth worm compost) was sterilized at 121 °C for 1 h in an autoclave and cooled before being applied. To analyze colonization by inoculated fungal strain, the plants (tomato, cucumber, soybean, corn, brinjal, mustard, cow pea, and onion) were uprooted gently from the experimental pots after 30 days of fungal spores for inoculated seeds. Root sample was washed with tap water, blotted using a blotting paper, and cut into 2 cm size approximately. The rot tips were dipped into 70% ethanol for few seconds. Then, it was surface sterilized for 3 min using NaOCl (0.5%) and washed with double-distilled water. The surface-sterilized root was washed with double-distilled water and plated on PDA medium to confirm the elimination of epiphytic organism from the root. Then, it was macerated with sterile double-distilled water using a pestle and mortar. Then, the sample was spread on PDA medium and amended with streptomycin salt and tetracycline at 0.05%. The growth of *P. formosus* MD12 was observed at 25 ± 2 °C after 5 days. Positive colonization was scored by determining the number of pieces of the root with growth of *P. formosus* MD12 [26]. To determine whether the growing entophytes were the ones initially inoculated; microscopic slides were prepared from mother plates (*P. formosus* MD12) and compared with the slides of root by morphological identification. 

### 2.6. Source and Maintenance of Nematode Culture

*M. incognita* eggs were initially extracted from the decayed brinjal root using 1% (*v*/*v*) sodium hypochlorite; then, it was poured onto mesh and washed repeatedly with tap water, and an excess amount of sodium hypochlorite was removed. The eggs were transferred to a clean container thatwas filled with sterile water. 

### 2.7. Egg Hatch Inhibition Assay by Cell Free Extract of Fungus

In vitro analysis was performed to evaluate the influence of cell-free extract of the selected fungus at various dilutions. The nematode egg concentration was adjusted to 200 numbers in one mL and was transferred to a plastic container with 20 mL cell-free fungal extract at various concentrations. To the control vials, 20 mL of distilled water was incorporated with 200 numbers of *M. incognita* eggs. 

### 2.8. Influence of Spore Suspensions on the Activity of M. incognita Eggs

The efficacy of conidial spore suspensions from *P. formosus* MD12 against *M. incognita* eggs was evaluated. The viable conidia were prepared at five different (1 × 10^4^, 1 × 10^5^, 1 × 10^6^, 1 × 10^7^, and 1 × 10^8^ CFU mL^−1^) concentrations. These were directly applied to a nematode egg by inoculating 1 mL of sterile double-distilled water containing 200 eggs in plastic containers with 20 mL of water. To the control, an equal amount of nematode egg was inoculated without spore suspension. Treatment was conducted for 96 h, and the results were observed for every 24 h. The non-hatched eggs were counted, and the percentage of egg hatch inhibition was analyzed [22].

### 2.9. Effect of P. formosus MD12 on Juveniles of Nematode

For determining the influence of *P. formosus* MD12 extract on *M. incognita* juveniles (stage II), the extract was added. The second-stage juvenile was extracted from the pot using a sieving and decanting technique. In addition, *M. incognita* juvenile was extracted from the roots of brinjal plants and placed in a vial containing sterile water. *M. incognita* juvenile (*n* = 50) was incubated with cell-free extract of *P. formosus*. To the control, double-distilled water was added with the *M. incognita* juvenile. It was incubated for 72 h, and every 24 h, alive and dead juveniles were counted using a light microscope [22].

### 2.10. Pot Experiment

Brinjal (*Solanum melongena*)(CO1 breed) seeds were collected from the market and used for cultivation. A total of 40 seeds were sown innine experimental pots, and a control pot containing soil collected from the agricultural field. Four seeds were used in each pot containing approximately 3 kg soil. Each pot was supplemented with tap water at 6 am and 6 pm daily. Then, newly developed plants were divided into two groups. To the first five pots, the cell-free supernatant was added at 2%, 4%, 6%, 8%, and 10% levels. In another four experimental pots, spore suspension (1 × 10^4^, 1 × 10^5^, 1 × 10^6^, and 1 × 10^7^ CFU mL^−1^) was added using a sterile micropipette. The culture supernatant and spores were introduced individually around the newly developing plants after 2 weeks in three holes (10 mm diameter). *M. incognita* juveniles (*n* = 500) were introduced in all experimental pots. Brinjal was grown for three months, and after three months of inoculation, it was uprooted from the experimental and control pots. Roots were cut into small pieces (1 cm size) and incubated in tap water, and the juvenile stage of nematodes was counted using a light microscope. The growth of shoot and root length (cm) was observed. A randomized experimental trial was made, and all experiments were performed in triplicates [4]. 

### 2.11. Enzyme Extraction

Brinjal leaves were collected from the experimental and control pots and cut into small pieces (<0.5 cm size). Four leaves were collected from each pot (one leaf from each plant). Then, one-gram leaf pieces were ground with 4.0 mL of phosphate buffer saline (pH 7.2, 0.1 M) using a glass homogenizer. After complete homogenization, it was centrifuged at 8000 rpm for 10 min. The clear supernatant was used as the sample for enzymeassay.

### 2.12. Peroxidase Activity 

Peroxidase (POD) activity of the sample was determined by a UV-visible spectrophotometer using guaiacol as a substrate. To the sample (0.1 mL), 1 mL buffer (sodium acetate buffer, pH 5.5), 0.2 mL guaiacol (1%, *w*/*v*), and 0.2 mL H_2_O_2_ (1%, *v*/*v*) were added. The mixture was incubated for 5 min at 25 °C, and the absorbance of the sample was read at 436 nm against reagent blank [27].

### 2.13. Polyphenoloxidase Activity

Polyphenoloxidase (PPO) activity of the sample was determined using a UV-visible spectrophotometer. To the sample (0.25 mL), 1 mL of sodium phosphate buffer (0.05 M) and 0.5 mL of substrate (brenzcatechin) was added. It was incubated for 30 min at 37 °C and the absorbance was measured at 410 nm [27].

### 2.14. Seed Germination Analysis

To analyze the non-toxic properties of the selected fungi, the brinjal seeds were immersed in sterile double-distilled water containing 1 × 10^4^ to 1 × 10^7^ fungal spores mL^−1^. To the control experiment, seeds were immersed only in double-distilled water. Seeds were soaked for 4 h and blotted. The seeds treated with spores were maintained at 27 ± 2 °C for one week. The percentage seed germination was analyzed. 

### 2.15. Data Analysis

Analytical experiments were performed in triplicate experiments. The pot experiment was performed in three different experiments with randomized trials. For the determination of enzyme activity, triplicate analyses were performed. Analysis of variance (ANOVA) was performed and significance was tested.

## 3. Results

### 3.1. Characterization of P. formosus MD12 and Density of Fungi in Soil and Rhizosphere Sample 

**Figure 1:**
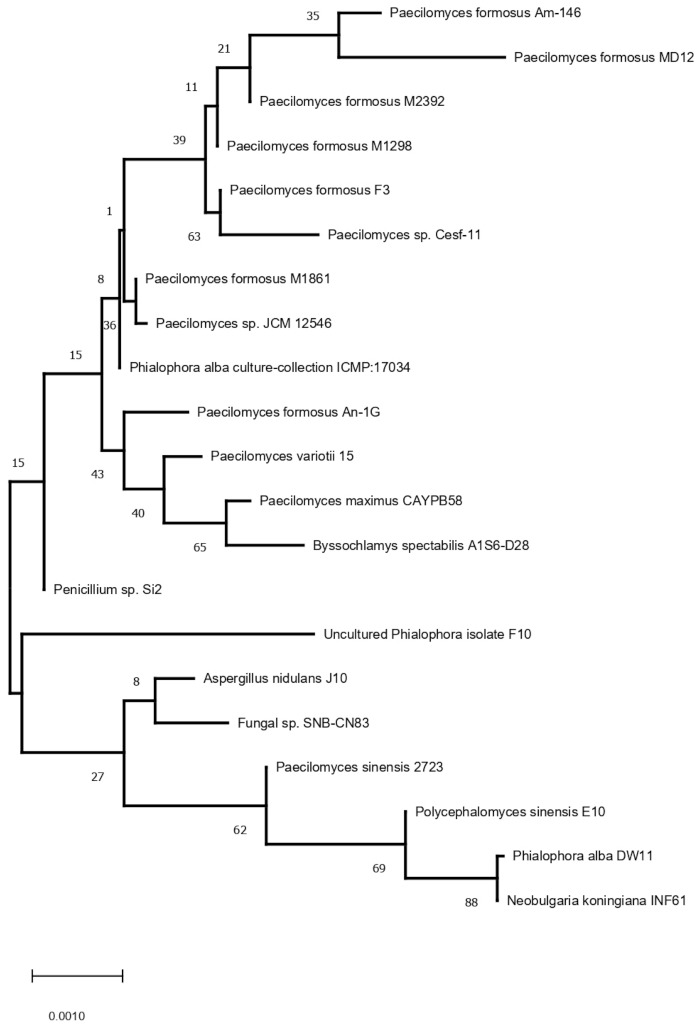
Phylogenetic relationships of *P. formosus* MD12 with closely species based on the Neighbor-Joining method.

The fungus strain MD12 was identified as *P. formosus* MD12 based on 18S rDNA gene sequence analysis. The phylogeny analysis of 18S rDNA gene sequence of MD12 with closely related sequence of blast results was performed followed by multiple sequence alignment. The ITS region was sequenced using ITS1 and ITS4 primers, and ITS sequences are generally conserved and show very little variation within species level; however, they vary between species level in a particular genus. Phylogenetic relationships of *P. formosus* MD12 with closely species described in Figure 1. *Paecilomyces* species play a significant role as an endophyte in various plants by providing several advantages for plant growth. *Paecilomyces* or its metabolites showed good biocontrol ability against the root-knot nematode and colonize in soil and the rhizosphere. The density of fungi varied in soil and the rhizosphere after *P. formosus* MD12 treatment. The *P. formosus* MD12 population was 6.3 ± 0.13 CFU g^−1^ in the pot plated with tomato and the population has increased in the rhizosphere (6.72 ± 0.41 CFU g^−1^). In the case of brinjal, the soil fungi concentration was 7.68 ± 0.13 CFU g^−1^, and an increased level of fungi was observed in the rhizosphere (8.37 ± 2.6 CFU g^−1^). Moreover, the fungi population in the rhizosphere of mustard, onion, and tomato was also enhanced. ANOVA revealed a significant variation of fungi in soil (F value: 134; *p* < 0.001) associated with plants and rhizosphere of plants (F value: 1207; *p* < 0.0001) (Table 1).

### 3.2. Endophytic Colonization of P. formosus MD12

The viability tests revealed the germination of conidia at various percentages for all selected strains. The seeds that were not inoculated with conidia suspension did not show any growth on PDA plates. The fungal strain was able to colonize all selected plants seeds. Moreover, the extent of colonization of the various plant seeds based on the types of seeds and fungal isolate. The mean colonization of tomato seeds by fungus was 6.3 ± 0.13% and cucumber was 5.8 ± 0.41, respectively. Seed inoculated with fungus showed colonization of 3.7 ± 0.58%, 2.1 ± 0.32%,7.68 ± 2.62%, 4.59 ± 1.93%, 1.76 ± 0.72%, and 3.2 ± 0.26%, respectively for soybean, corn, brinjal, mustard, cow pea, and onion seeds, respectively. 

### 3.3. Endophytic Root Colonization of P. formosus MD12 and Other Fungi in Experimental Plants

The densities of *P. formosus* MD12 and other endophyte populations in plants are described in Table 2. Tomato, cow pea, and onion showed a minimum of colonized *P. formosus* MD12 and other fungi. Among the plant species, brinjal supported maximum colonization in the root, and the colony count was 3.67 ± 0.06 CFU g^−1^. In corn, the population of endophytic fungus *P. formosus* MD12 was 2.86 ± 0.72 CFU g^−1^, and the other fungal population increased considerably (6.2 ± 1.2 CFU g^−1^). The endophytic root colonization of *P. formosus* MD12 and other fungal strains in a greenhouse condition was described in Table 2. *P. formosus* MD12 from the plants (F value: 5.58; *p* < 0.01) and other fungi population (F value: 17.7; *p* < 0.003) increased significantly. 

### 3.4. Influence of P. formosus MD12 Supernatant on Inhibition of Egg Hatching

**Figure 2:**
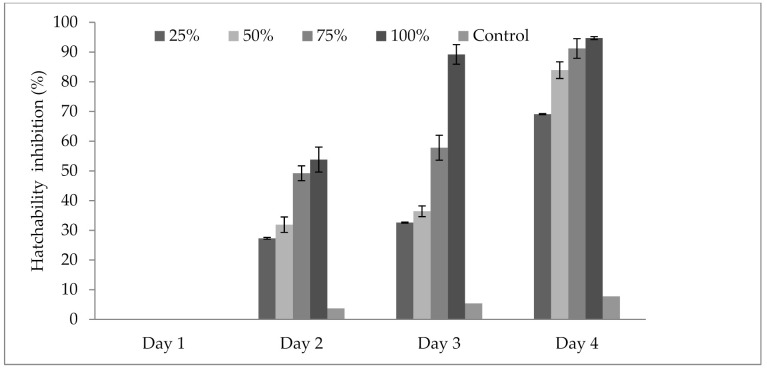
Egg hatchability inhibition of *Meloidogyne incognita* at various concentrations of culture extract from *P. formosus* MD12 after 96 h exposure. Error bar represents standard deviation.

The results in Figure 2 revealed that the culture supernatant of *P. formosus* MD12 affected the hatching ability of *M. incognita* eggs when exposed for 4 days. The hatching ability was compared with the control experiment at various concentrations (25–100%) and also at maximum incubation times (up to 4 days). The maximum egg hatchability inhibition (94.7 ± 6.2%) was obtained at 100% concentration of extract and was statistically significant. The other dilutions of *P. formosus* MD12 extract showed minimum egg inhibition. Egg inhibition was affected by incubation days (F value: 21.8; *p* = 0.001) and also concentration of culture supernatant (F value: 8.3; *p* = 0.01). Moreover, no significant difference was observed between Day 3 and Day 4 incubation at 100% supernatant (F value: 0.023; *p* = 0.977). 

### 3.5. Influence of P. formosus MD12 Spores on Egg Hatching

**Figure 3:**
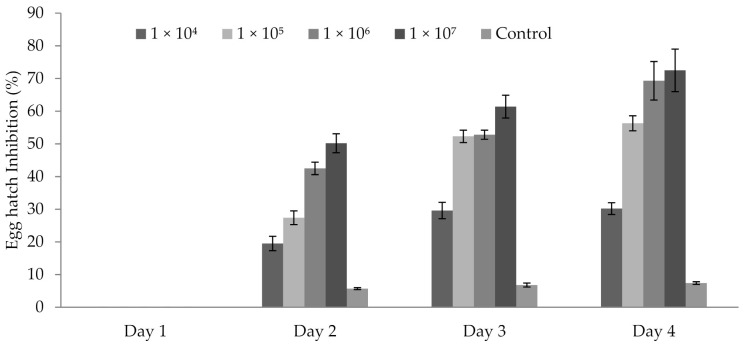
Egg hatch inhibition of *M. incognita* egg incubated at various concentrations of *P. formosus* MD12 spore after 96 h exposure. Error bar represents standard deviation.

*P. formosus* MD12 spores inhibited the egg hatching ability of *M. incognita* egg after 48 h. After 24 h, no inhibitory activity was observed. Egg hatch inhibition was 19.5 ± 3.2% at 1 × 10^5^ mL^−1^ spore concentration after 2 days; however, it improved after Day 3 (29.6 ± 1.9%) and Day 4 (30.2 ± 12.3%). At 1 × 10^6^ mL^−1^ spore concentration, inhibition was 27.4 ± 2.1%, 52.3 ± 1.9% and 56.3 ± 2.4%, respectively after Day 2, Day 3, and Day 4. The same trend was observed in 1 × 10^7^ mL^−1^ spore. In this experiment, treatment time (day) (F value: 17.9; *p* < 0.001) and spore concentration (F value: 24.6; *p* < 0.001) significantly affected egg hatching percentage (Figure 3).

### 3.6. Influence of P. formosus MD12 Supernatant on Juvenile Mortality

**Figure 4:**
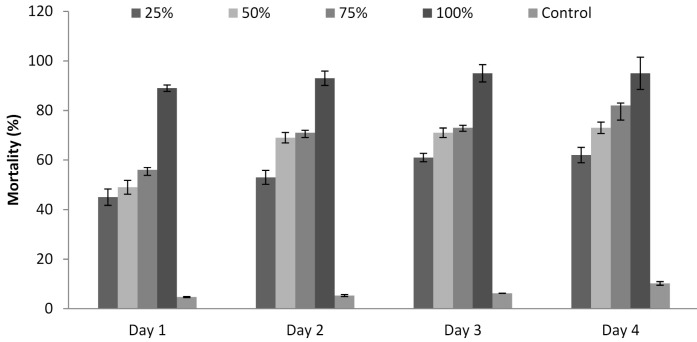
Influence of *P. formosus* MD12 extract on the mortality of *M. incognita* juvenile stage II under in vitro experimental trials. Error bar represents standard deviation.

The culture supernatant of *P. formosus* MD12 affected the development of *M. incognita*, and the mortality rate was recorded for 96 h. Mortality (%) was maximum at 96 h treatment (95 ± 6%) and lower at least incubation time (89 ± 3.4%) (24 h). Mortality was 62 ± 3.1%, 73 ± 2.3%, and 82 ± 5.9%, respectively when the juveniles were treated with 25%, 50%, and 75% supernatant after 4 days. Mortality was less after Day 1, and it increased continuously up to Day 4. ANOVA showed that the incubation time (F value: 13.9; *p* = 0.001) and concentration of the supernatant (F value: 55.2; *p* < 0.001) significantly induced juvenile mortality (Figure 4).

### 3.7. Effect of Fungal Spore Suspension and Supernatant on the Reduction of Nematode Population in Pot and Plant

The number of juvenile nematodes in root, soil, and egg masses was the clean indicators to study the efficacy of the analyzed fungus compared with the control pot. The fungal spore has the potential to reduce the nematode population significantly. At maximum concentration (1 × 10^7^ mL^−1^) of spore suspension, there were reductions of 71.6 ± 3.3% of the nematode population in the soil, 60.7 ± 2.2% of the population from the root, and 63.6 ± 2.4 of the egg mass compared with the control. The ANOVA test revealed a significant reduction of nematode population at various concentrations of spores (F value: 44; *p*< 0.0001), and the nematode population significantly varied among root, shoot, and egg masses (F value: 22.3; *p* = 0.001) (Table 3a). The number of juvenile nematodes decreased in roots and soil, and the number of egg masses decreased in the experimental pot compared with the control. The nematode population (%) was reduced for all selected concentrations of fungal supernatant compared with the control. The culture supernatant was applied at the 2% level, which showed the reduction of nematode population in roots (40 ± 2.1%), soil (51.1± 3.8%), and egg masses (42.9 ± 4.2%), respectively. At 10% supernatant, the nematode population was 72.4 ± 2.2%, 77.9 ± 2.5%, and 73.2 ± 1.5% in roots, soil, and egg masses, respectively. The nematode population was influenced by various concentrations of culture supernatant (F value: 48; *p* < 0.0001), and the population of nematodes varied among root, soil, and egg masses (F value: 17; *p* < 0.001) (Table 3b). 

### 3.8. Effect of P. formosus MD12 Culture Supernatant and Spores on Brinjal Plant Growth

**Figure 5:**
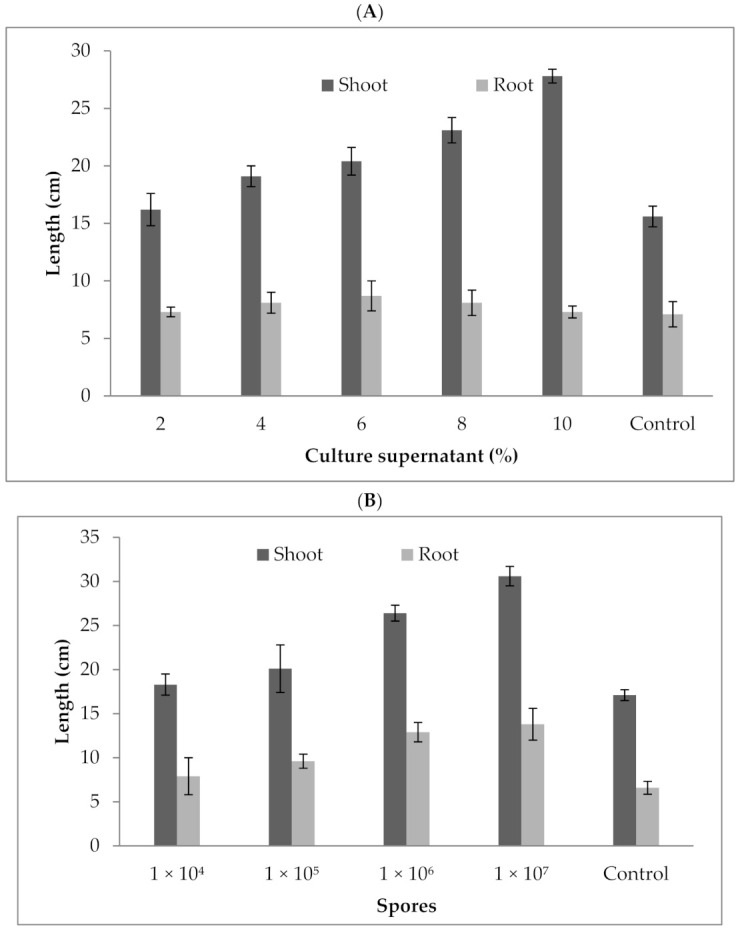
Influence of *P. formosus* MD12 culture supernatant (**A**) and spores (**B**) on shoot and root growth in brinjal. Error bar represents standard deviation.

Tbe *P. formosus* MD12 culture supernatant enhanced shoot and root length in brinjal. At 2% treatment, the shoot length was 16.2 ± 1.4 cm, and it increased to 27.8 ± 0.6 cm at 10% treatment. Likewise, the root length was 7.3 ± 0.43 cm at 2% supernatant treatment, and it reached 14.2 ± 0.52 cm at 10% treatment. Brinjal growth increased significantly after the plant was treated with spores. Shoot length and root length were 30.6 ± 1.1 cm and 13.8 ± 1.8 cm, respectively, at 1 × 10^7^/mL spore concentration. The shoot length was improved by 78%, and the root length was enhanced more than two fold compared with the control. The culture supernatant at various concentrations (2–10%) significantly improved shoot (F value: 16.2; *p* = 0.01) and root length (F value: 210; *p* = 0.0001). As for root and shoot length, maximum growth was achieved at 10%cell-free extract treatment than control. The root (F value: 9.87; *p* = 0.02) and shoot length (F value: 98; *p* < 0.001) was improved significantly compared with the control (17.1 ± 1.1 cm and 6.59 ± 1.8) (Figure 5a,b). The culture supernatant at various concentrations (2–10%) significantly improved the shoot (F value: 16.2; *p* = 0.01) and root length (F value: 210; *p* = 0.0001). 

### 3.9. Influence of Fungal Spores on Peroxidase and Polyphenoloxidase Activity

**Figure 6:**
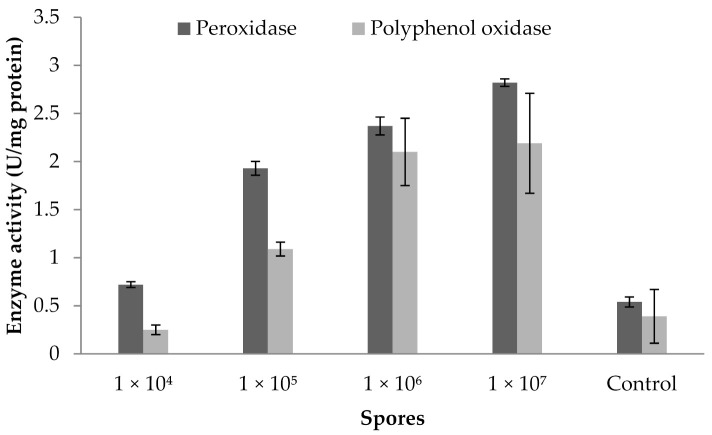
Peroxidase and polyphenoloxidase activity of plant inoculated with various concentrations of spores. Error bar represents standard deviation.

The peroxidase and polyphenoloxidase activity of a brinjal plant cultivated in greenhouse condition is described in Figure 6. The findings show the influence of the use of a biocontrol agent in a greenhouse environment. Figure 6 clearly reveals that the fungal spore treated at 1 × 10^7^ mL^−1^ concentration showed increased POD activity (2.82 ± 0.039 U mg^−1^ protein). PPO activity also improved considerably (2.19 ± 0.52 U mg protein^−1^) at this concentration. In addition, the application of spores reduced POD and PPO activity at 1 × 10^4^ mL^−1^ concentrations. ANOVA revealed that fungus spores at various concentrations enhanced the POD activity (F value: 58; *p* = 0.003) and PPO activity in brinjal leaves (F value: 20.8; *p* = 0.01). 

### 3.10. Influence of Fungal Spores on Seed Germination

**Figure 7:**
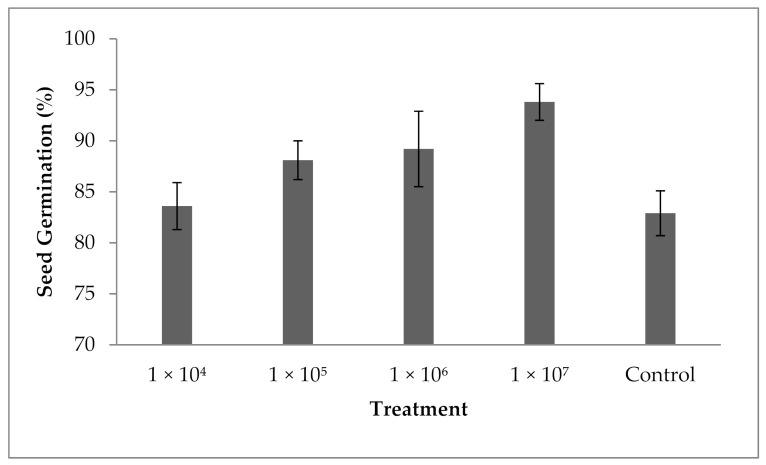
Influence of *P. formosus* MD12 on the germination of brinjal under laboratory condition. Error bar represents standard deviation.

The results revealed that spore suspension at various concentrations enhanced the percentage of seed germination. The percentage of seed germination was 82.9 ± 2.1% in the control and the seed germination percentage was enhanced in the experiment containing 10^4^ spores mL^−1^. A significant difference in the seed germination percentage has been achieved in all tested spores suspensions; however, increased concentrations of spores improved the seed germination percentage, and the variation in seed germination was statistically significant (F value: 13.4; *p* = 0.001). At 1 × 10^7^ spores mL^−1^ concentration, seed germination was improved (93.8 ± 2.6%). The present finding revealed that *P. formosus* MD12 has a significant impact on the germination of brinjal seeds in tested spores concentrations (Figure 7).

## 4. Discussion

In vitro experiments and in vivo pot trials proved that the tested fungus either as spore or supernatant affected the hatching ability of nematode parasites and the survival of juveniles at various degrees according to the culture supernatant, spore concentration, and contact time. The percentage of nematode larval mortality and inhibition of nematode parasite were according to the concentration of culture supernatant, which agreed with the results of Zhao et al. [28]. Pathogenic fungi produced toxins such as destruxins and cyclopeptides, and these toxins played a significant role in pathogenicity. The selected strain, *P. formosus* MD12, inhibited egg-hatching ability and induced mortality in second-stage *M. incognita* juvenile. The culture supernatant and fungus spore induced >80% mortality at high concentration of spore (>1 × 10^4^ spores mL^−1^) and supernatant (>75%). The present findings revealed that the fungus culture supernatant and spore suspension of *P. formosus* MD12 under greenhouse conditions suppressed reproductive parameters and enhanced the growth of brinjal. It was previously reported that the fungus, *Paecilomyces* sp., showed activity against nematode parasite. The penetration of fungi hyphae on the cuticle is one of the mechanisms to destroy *M. javanica* [29]; moreover, the penetration of toxins into egg embryos including leucino toxins and other nematicidal compounds has been reported [20]. Successful nematodes control in vegetables is based on the colonization, proliferation, and survival of mycelia that are able to attack nematodes. The serine protease synthesized by *Paecilomyces* sp. induced the deformation of nematode eggs and induced death in juveniles [30]. 

The culture extracts of *T. viride*, *F. oxysporum*, *A. niger*, and *A. nidulans* killed about 50% of second-stage juveniles. Moreover, culture extracts from *P. chlamydosporia*, *P. lilacinus*, *F. chlamydosporium*, *C. oxysporum*, and *C. aubense* caused decreased mortality, and the culture extract of *F. solani* did not show any nematocidal activity. Fungi produce toxic compounds or either parasitizes nematodes, which are highly harmful to various parasitic nematodes. *A. terreus* and *A. strictum* showed biological control properties against *M. incognita* and reported that *A. strictum* not only parasitize eggs but also kill the juvenile stage of parasites [31]. The present finding indicates its potency as a promising biocontrol agent on root-knot nematodes and subsequently on the plant growth-promoting property in Brinjal, which is one of the important vegetable crops in Asia. The present finding proved that the culture extract and spores improved the root length and shoot length in Brinjal. Fungal toxins, antibiotics, and various toxic substances secreted by fungal species as well as direct interactions might be highly responsible for the mortality of second-stage juvenile. Recently, Huang et al. [32] tested the efficacy of *Paecilomyces lilacinus* and *Syncephalastrum racemosum* to treat root-knot nematode (*Meloidogyne incognita*) cucumber plants. The combination of *Paecilomyces lilacinus* and *Syncephalas trumracemosum* were found to be suitable for the treatment of root-knot disease. The nutritional and protective properties of applied fungus make it a promising biological agent for the nematode involved in root-knot diseases mainly in an organic farming system, where plant disease control and nutrition are the limiting factors. 

The root-knot nematode is one of the major nematodes that cause serious damage in the production of various economically important agricultural crops and vegetables. This parasite causes about 5% of worldwide crop loss. *M. incognita* caused more than 25% damage in tomato crop [33]. Fungi from the genus *Trichoderma* spp. have been effective against *M. incognita* [34]. The selected fungus, *P. formosus* MD12, could colonize the rhizosphere of brinjal plant and improved growth and showed inhibitory activity against nematode parasite. Soil amended with fungal spores enhanced plant growth in greenhouse condition. This result indicates that *P. formosus* MD12 adds additional growth promoters to the crop plants. The selected nematophagous fungus colonizes on the brinjal plant and proliferates in the rhizosphere of brinjal. In crop plants, the successful biocontrol of nematodes is mainly based on the establishment, proliferation, and survival of fungi through soil as spores capable of attacking nematodes [35]. The present result revealed increased colonization after inoculums of fungal spores. The number of spores improved after 72 h, which revealed the colonization and proliferation of fungal spores on brinjal plants. Fungal bioagents either produce toxins to plant nematode parasites or parasitize on nematodes [36]. The fungus, *P. formosus* MD12 isolated from brinjal, showed an inhibitory effect on *M. incognita.* The fungi such as, *Paecilomyces lilacinus*, *Trichoderma harzianum* produced various toxins that affected the second-stage juveniles of *Meloidogyne incognita* in brinjal [37]. *P. formosus* MD12 spores or culture supernatant treated in a pot decreased nematode load and infection. This is mainly attributed to the toxins produced by the fungus and the penetration of fungal hyphae on nematode parasite directly. *P. formosus* MD12 has the potential to produce cytokinin-like compounds as well as indol-3-acetic acid. The mixing of fungal spores with brinjal seeds activated the defense enzyme system and improved the resistance in plants. The life cycle of nematode parasite and the mechanism of the secondary metabolite producing fungi were not analyzed in this study. However, the mechanism and mode of action have been described in certain cases. The J2 stage of *M. incognita* is an obligate biotroph that mainly depends on its host species for survival and growth. These mainly include the consumption of its reserve lipid prior to finding a host species or starvation [38]. In a study, Holscher et al. [39] reported that when *Radopholussimilis* (nematode parasite) was treated with anigorufone (nematocidal compound), oil droplets formed inside the body of the nematode around the anigorufone compound. Plant parasitic nematodes consist of four larval stages and the adult female and male. *M. incognita* completes the first cycle within the egg, and in the second stage, it infects plant root and root-knots were formed and completed their remaining stages within 8 weeks [40,41]. El-Sheikh et al. [42] isolated *Paecilomyces* sp. ZB for the production of gibberellic acid and indol-3-acetic acid. Govindappa et al. [43] reported maximum peroxidase activity in plants compared with control plants when treated with *Bacillus subtilis*, *Trichoderma harzianum*, and *P. fluorescens.* These biocontrol agents were involved in the generation of antimicrobial agents, producing free radicals involved in the process of lignification. PPO activity in the plant was enhanced because it catalyzes the final step of production of lignin and various oxidative phenols.

## 5. Conclusions

The application of *P. formosus* MD12 not only affects the survival rate of the root-knot nematode but also improves the growth of brinjal. The presence of *P. formosus* MD12 could effectively antagonize other fungi from the soil and rhizosphere of brinjal. The present study reveals the use of *P. formosus* MD12 as a biocontrol agent to control *M.incognita* population in brinjal.

## Figures and Tables

**Table 1:** Fungi population density in the soil and rhizosphere in various plant species in a greenhouse.

	Fungi (CFU g^−1^)	
Plants	Soil	Rhizosphere
Tomato	6.3 ± 0.13	6.72 ± 0.27
Cucumber	5.8 ± 0.41	4.3 ± 0.86
Soybean	3.7 ± 0.58	3.56 ± 1.1
Corn	2.1 ± 0.32	1.92 ± 0.5
Brinjal	7.68 ± 2.62	8.37 ± 2.6
Mustard	4.59 ± 1.93	5.2 ± 0.42
Cow pea	1.76 ± 0.72	0.41 ± 0.02
Onion	3.2 ± 0.26	3.51 ± 0.86

**Table 2:** Endopohytic root colonization of *P. formosus* MD12 and other fungal strain in a greenhouse.

	Fungi (CFU g^−1^)	
Plants	*P. formosus*	Other Fungi
Tomato	0.39 ± 0.0	1.9 ± 0.07
Cucumber	0.34 ± 0.02	0.69 ± 0.05
Soybean	2.34 ± 0.18	3.7 ± 0.92
Corn	2.86 ± 0.72	6.2 ± 1.2
Brinjal	3.867 ± 0.82	8.9 ± 1.8
Mustard	2.28 ± 0.65	3.5 ± 1.1
Cow pea	1.09 ± 0.52	5.3 ± 0.2
Onion * Control	0.21 ± 0.03 0.13 ± 0.02	2.6 ± 1.3 0.52 ± 0.27

*P. formosus* MD12 and other fungi population were expressed as mean ± standard deviation. * Brinjal seed soaked with sterile water was considered as control. *P. formosus* MD12 and other fungi showed maximum colonization in Brinjal root; hence, brinjal seed without any inoculum was tabulated as control.

**Table 3:** Reduction of nematode population in soil and root, and reduction of egg mass.

(a)			
Spore Suspension (CFU mL^−1^)	Reduction of Nematode (%)		
	Root	Soil	Egg Masses
1 × 10^4^	47 ± 1.9	53.1 ± 2.8	40.9 ± 3.2
1 × 10^5^	51 ± 2.6	60.2 ± 3.3	52.7 ± 1.6
1 × 10^6^	58.4 ± 1.1	67.2 ± 2.7	60.3 ± 2.7
1 × 10^7^ Control	60.7 ± 2.2 4.1 ± 0.3	71.6 ± 3.3 0.8 ± 0.42	63.6 ± 2.4 0 ± 0
**(b)**			
**Culture Supernatant (%)**	**Reduction of Nematode (%)**		
	**Root**	**Soil**	**Egg Masses**
2	40 ± 2.1	51.1 ± 3.8	42.9 ± 4.2
4	43 ± 2.5	61.3 ± 2.3	56.7 ± 2.7
6	59.2 ± 1.5	69.2 ± 2.2	68.2 ± 3.1
8	63.8 ± 3.3	76.8 ± 3.7	69.2 ± 1.9
10 Control	72.4 ± 2.2 2 ± 0.1	77.9 ± 2.5 0.48 ± 0.16	73.2 ± 1.5 4.1 ± 0.7

(**a**). by spore suspension from *P. formosus* MD12. (**b**). by culture supernatant from *P. formosus* MD12.

## Data Availability

The data supporting the findings of this study are presented within this article. Any additional data available on request to the author.
